# Recent breeding programs enhanced genetic diversity in both desi and kabuli varieties of chickpea (*Cicer arietinum* L.)

**DOI:** 10.1038/srep38636

**Published:** 2016-12-16

**Authors:** Mahendar Thudi, Annapurna Chitikineni, Xin Liu, Weiming He, Manish Roorkiwal, Wei Yang, Jianbo Jian, Dadakhalandar Doddamani, Pooran M. Gaur, Abhishek Rathore, Srinivasan Samineni, Rachit K. Saxena, Dawen Xu, Narendra P. Singh, Sushil K. Chaturvedi, Gengyun Zhang, Jun Wang, Swapan K. Datta, Xun Xu, Rajeev K. Varshney

**Affiliations:** 1 International Crops Research Institute for the Semi-Arid Tropics (ICRISAT), Hyderabad, India; 2 BGI - Shenzhen, China; 3 All India Coordinated Research Project (AICRP) on Chickpea, Indian Council of Agricultural Research (ICAR), New Delhi, India; 4 Indian Institute of Pulses Research (IIPR), Indian Council of Agricultural Research (ICAR), Kanpur, India; 5 Visva-Bharati, Santiniketan, India

## Abstract

In order to understand the impact of breeding on genetic diversity and gain insights into temporal trends in diversity in chickpea, a set of 100 chickpea varieties released in 14 countries between 1948 and 2012 were re-sequenced. For analysis, the re-sequencing data for 29 varieties available from an earlier study was also included. Copy number variations and presence absence variations identified in the present study have potential to drive phenotypic variations for trait improvement. Re-sequencing of a large number of varieties has provided opportunities to inspect the genetic and genomic changes reflecting the history of breeding, which we consider as breeding signatures and the selected loci may provide targets for crop improvement. Our study also reports enhanced diversity in both desi and kabuli varieties as a result of recent chickpea breeding efforts. The current study will aid the explicit efforts to breed for local adaptation in the context of anticipated climate changes.

Chickpea (*Cicer arietinum* L.) is one of the most important annual pulse crops cultivated by resource poor farmers across the globe. Besides being the rich source of human dietary proteins, it improves the soil health through symbiotic nitrogen fixation. Globally it is cultivated on over 13.2 Mha with an annual production of 13.1 million tons^1^ and productivity is less than 1 t/ha, much less than estimated potential of 6 t/ha under optimum growing conditions. Unpredictable global climate changes^2^ coupled with ever increasing human population led to growing hunger and increased malnutrition. With limited arable land, attaining sustainable food production is a challenge before scientific community. Breeding pursuits in the past to enhance production although led to development of improved cultivars, genetic bottlenecks and subsequent founder effects during domestication resulted in narrow genetic base, especially in crops like chickpea^3^. As a result, susceptibility of the crop has been aggravated to several biotic and abiotic stresses and production potential has been seriously hampered. To overcome these constraints, genomic approaches have been deployed in recent years to understand the genetic basis of such complex quantitative traits and for trait improvement in chickpea^4^. Several superior lines with enhanced drought tolerance and resistance to Fusarium wilt (FW; caused by *Fusarium oxysporum* fsp *ciceris*) and Ascochyta blight (AB; caused by *Ascochyta rabiei*), have been developed^5^,^6^.

Naturally occurring genetic variation is a valuable source of alleles that can be deployed for trait improvement. In this direction, earlier efforts to understand the genetic diversity trends using pedigree information demonstrated difficulty in drawing general conclusions^7^, although narrow genetic base of legume varieties was evident from the pedigree information^8^. Nevertheless, several studies employing molecular markers in different crop species indicated reduction of diversity followed spatial and sometimes temporal trends^9^,^10^,^11^. More recently, a meta-analysis of genetic diversity trends in twentieth century crop cultivars like maize (*Zea mays*), wheat (*Triticum aestivum*), barley (*Hordeum vulgare*), soybean (*Glycine max*), pea (*Pisum sativum*) etc., by van de Wouw and colleagues^12^ indicated an increase in diversity in varieties after 1970s or no major reduction in diversity of varieties was observed. In general, limited number of markers, that might have not represented the genome of the given crop species, were used in these studies. Next-generation sequencing (NGS) technologies have enabled understanding the genome dynamics and genome architecture of several important crop plants^13^ including chickpea^14^. Further, in order to understand untapped genetic potential available for crop improvement in a species, some germplasm lines have also been re-sequenced using whole genome re-sequencing (WGRS) approach^15^,^16^,^17^,^18^,^19^.

Over 350 chickpea varieties with desirable traits like early maturity, tolerance to stresses, have been released globally^20^. Understanding the diversity at genome level in case of the varieties will enable utilization of alleles gained or lost over decadal periods, to address the future challenges and develop climate smart and resilient chickpea varieties. This study reports sequencing of 100 varieties and analyzes 129 varieties after combining the sequence data generated in this study together with 29 varieties generated earlier. In brief, the study improves current understanding of temporal dynamics of chickpea diversity by examining the genome-wide variations in 129 varieties. In addition, we report the genomic regions with narrow genetic diversity and the regions rich in novel alleles and haplotypes that can be targeted for trait improvement and broadening the genetic base of chickpea.

## Results

In order to gain insights into the temporal and genome diversity trends, we re-sequenced 100 chickpea varieties released between 1948 and 2012 on Illumina HiSeq 2000 using WGRS approach. In addition, re-sequence data available on 29 chickpea varieties/breeding lines/cultivars from our earlier study^14^ was used in this study. Thus, a total of 129 chickpea varieties, comprising 88 desi and 41 kabuli, released in 14 countries across the globe were analysed in this study.

### Salient features of varieties

**Figure 1:**
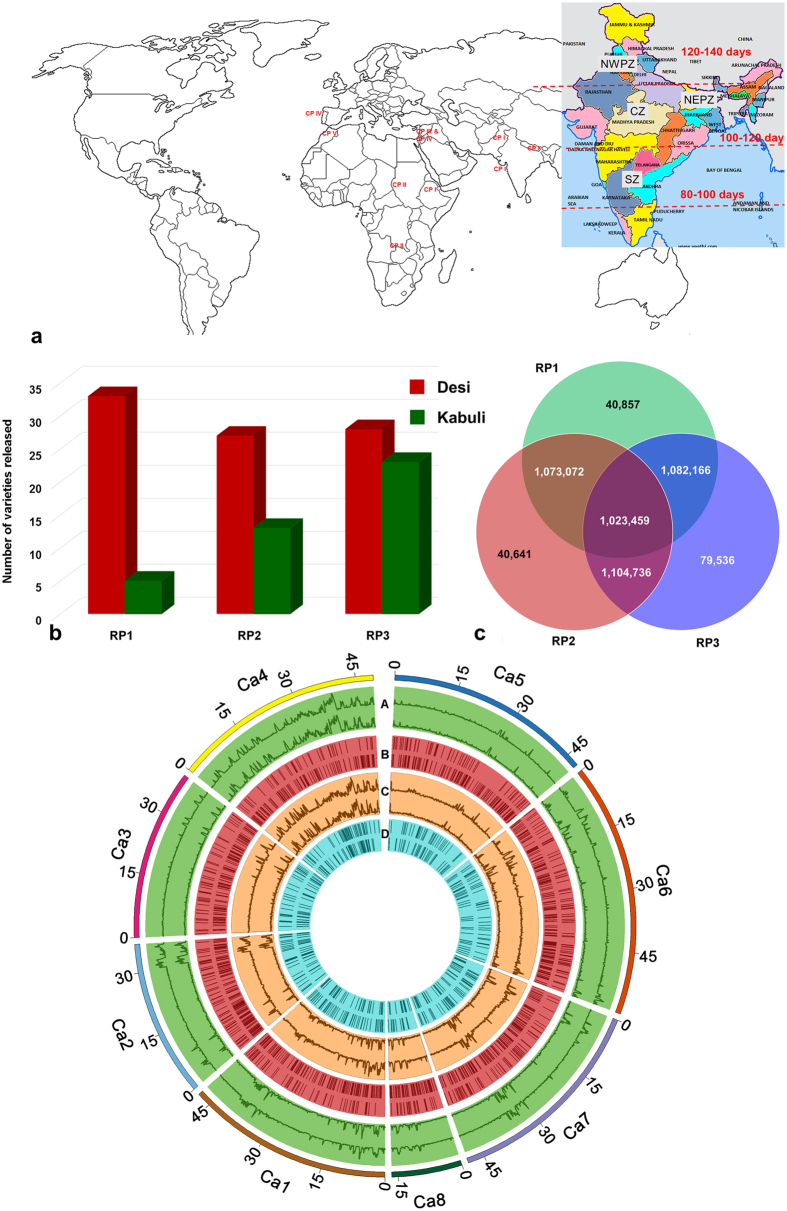
Global distribution of 129 chickpea varieties and genome-wide variations. (**a**) A set of 129 varieties released in 14 important chickpea growing countries across the globe. The varieties used in the study encompass all five global chickpea research domains (CPI, CPII, CPIII, CPIV and CPV) as well as all chickpea growing zones in India (North Western Plain Zones, NWPZ; North Eastern Plain Zones, NEPZ; Central Zone, CZ and Southern Zone, SZ). Information on chickpea research domains across the globe and growing zones in India is taken from public domain, while the maps were drawn using licensed ArcGIS software 10.3 for Desktop. (**b**) For understanding the temporal trends in diversity, and to avoid biased estimates 129 varieties are grouped into three time periods (i) varieties released in RP1 (before 1992), (ii) varieties released in RP2 (between 1993 and 2002), (iii) varieties released in RP3 (after 2002). An increase in number of kabuli varieties after 2002 can be observed. (**c**) Venn diagram represents both shared SNPs as well as unique SNPs among varieties released in different time periods. Large number of unique SNPs in varieties released after 2002 indicates an enhancement in diversity in the primary gene pool as a result of recent breeding programs. (**d**) Circos diagram represents the genome - wide variations among 129 varieties. Eight pseudomolecules are traversing from out to in: (A) SNP density (green), upper half desi and lower kabuli, (B) copy number variations, (C) Indel density and (D) presence and absence variations.

To avoid biased estimates of diversity and inaccurate conclusions owing to small sample number of varieties over one decadal period to the other, while assessing temporal trends in diversity, we grouped 129 varieties into three temporal groups: (a) Varieties released before 1993 (here after refereed as RP1); (b) Varieties released between 1993 and 2002 (RP2); and (c) Varieties released after 2002 (RP3). Number of varieties released in each of the 14 important chickpea growing countries are shown in Fig. 1a. These 129 varieties represent all five global chickpea research domains identified for chickpea improvement at ICRISAT based on critical criteria like latitude, length of growing period, temperature and soil type^21^. Further, these varieties also represent all chickpea growing zones including North Western Plain Zone (NWPZ; long duration varieties with 120–140 days to maturity), North Eastern Plain Zone (NEPZ; medium duration varieties with 100–120 days to maturity), Central Zone (medium duration varieties with 100–120 days to maturity) and Southern Zone (short duration varieties with 85–100 days to maturity) in India (Supplementary Table 1).

Based on pedigree, it was evident that 103 varieties were derived from conventional crossing programs, while 16 were direct selections from local germplasm/farmer fields and 4 were as a result of mutation breeding (Supplementary Table 1). Of 38 varieties in RP1, 6 (15.7%) varieties had JG 62 and 4 (10.52%) varieties had L 550 in their genetic background. While K 850, Pusa 256, JG 62 and L 550 were extensively used in developing varieties in RP2. In addition to JG 62, L 550 and K 850, ICCV 10 was also frequently used in developing varieties released in RP3. Release of kabuli varieties increased after 1992 (Fig. 1b). Further, greater proportion of varieties besides high yielding, were resistant to biotic stresses like FW, AB, pod borer (*Helicoverpa armigera*) and include short, medium and long duration in terms of maturity.

### Distribution of genome-wide SNPs and Indels

We obtained 8.36 billion clean reads after filtering 8.67 billion raw reads, on the basis of Q20 score. On aligning clean reads to draft genome assembly using SOAPaligner^22^, we obtained 7.59X average mapping depth and 88.6% coverage in case of unique alignment, while all alignment provided ~10.6X mean depth and 96% chickpea genome coverage (Supplementary Table 2). We identified a total of 1.37 million SNPs using SOAPsnp^23^ at an average one SNP per every 382 bases (Table 1). A total of 1.19 million SNPs were common among all three release periods (Fig. 1b). However, the number of unique SNPs were the highest (79,536) in case of RP3 varieties. Although the number of desi and kabuli varieties released were comparable, unique SNPs were higher in the case of desi varieties of RP3 (Supplementary Table 3; Fig. 1c). On an average majority of the SNPs (87.86%) were identified in the inter-genic regions followed by intronic (12.4%) and exonic (~3.3%) regions (Supplementary Table 4). Among SNPs in exonic regions, 56.7% were missense, 1.65% were nonsense and 41.58% were silent mutations (Supplementary Table 5). We identified large number of missense SNPs in case of Sona (ICCV 88202), a desi variety released in 1988 for cultivation in Western Australia (Supplementary Table 5). The highest number (257,604) of homozygous SNPs were identified in RAU 52, a medium duration variety released in Bihar in 1985, while the highest number (283,604) of heterozygous SNPs were identified in the case of ILC 3279, AB resistant kabuli variety released in 1988 in Algeria (Supplementary Figure 1).

We also observed large variation in number of SNPs and their distribution on eight pseudomolecules (Ca1 to Ca8) both in desi and kabuli varieties (Fig. 1d). Among eight pseudomolecules, Ca4 has the highest number of SNPs in both desi and kabuli varieties irrespective of period of release (Supplementary Table 6). Nevertheless, the SNP density in desi varieties was higher as compared to kabuli varieties (Supplementary Figure 2). We further classified the SNPs into synonymous (~56.2%), non-synonymous (~41.0%) and others (2.7%) based on their effect on genes (Supplementary Table 4). The dN/dS ratio varied between 1.12 and 1.75 with an average of 1.45, indicating positive selection at a genetic locus (Supplementary Figure 3).

In addition to SNPs, we identified small insertions and deletions called “Indels” (1–10 bp) by allowing gaps while aligning/mapping reads to the reference genome using SOAPindel^24^. Among 151,440 Indels, 51.14% (77,446) insertions and 48.86% (73,994) deletions were identified in non-coding regions of genome and <1% in coding region of the genome (Table 1 and Supplementary Tables 6 and 7). Among Indels, one base pair Indels were the most abundant in the genome as well as CDS regions (Supplementary Figure 4). Further, the proportion of 3 bp Indels, called as non-frameshift Indels, was high in coding regions compared to the other Indels. We observed a greater proportion of deletions in CDS region (~52%), while the proportion of insertions were high at whole genome level (Supplementary Table 8). Number of Indels, however, were higher in the case of RP1varieties followed by RP3 and RP2. Yezin 3 (ICCV 2), an early maturing kabuli variety released in 1983 for cultivation in Maharashtra and Andhra Pradesh, had the maximum number of Indels (31,924); while CDC Luna, another early maturing kabuli variety released in Canada in 2002 had minimum number of Indels (5,055). Interestingly, the average yield per hectare of CDC Luna (20 Q/ha) is comparatively higher than Yezin 3 (13–15 Q/ha).

### Structural variations in genes associated with agronomically important traits

Genome-wide structural variations like copy number variations (CNVs) and presence absence variations (PAVs) are common features of plant genomes and play an important role in contributing phenotypic variation and also contribute to genetic diversity of their genomes^25^. Based on mapping depth of each base to the reference genome, we identified a total of 3,822 CNVs across all 129 varieties (Supplementary Table 9). Number of CNVs in individual varieties varied from 541 to 1,508 ranging in size from 2.66–6.89 Mb. Unlike SNPs, Indels and PAVs that were abundant on the Ca4, CNVs were identified in large numbers both in genomic and CDS regions on the Ca6 (Supplementary Table 6). Nevertheless, no significant difference in number of CNVs was observed among varieties of RP1 (2,315 CNVs) and varieties of RP2 (2,318 CNVs). However, we observed significantly high number of CNVs (3,511) in varieties released in RP3 (Table 1). Of 765 CNVs in coding regions, 520 were in more than one variety and 145 were unique, while 12 CNVs were present in all 129 varieties. A total of 616 genes were found affected by CNVs, which accounts for 2.21% of total genes in chickpea. Through the gene ontology (GO), pathway analysis and comparison of CNV genes among different varieties, we found evidence that CNVs involving 33 genes are associated with disease resistance or defense response (Supplementary Table 10). The chromosome location of the CNVs overlapping R genes indicated the tendency clustering of these gene families in the genome, with the distal ends of Ca2 and Ca6 containing the highest number of variants. Further, GO-term enrichment analysis revealed that genes affected by CNVs are enriched for genes belonging to category of response to stimulus (Supplementary Figure 5). Similarly, earlier studies also indicated that CNVs have implications on phenotypic expression for instance, enhanced resistance to soybean cyst nematode (*Heterodera glycines*) with CNVs at *Rhg1* locus^26^, increased tolerance to boron-toxicity in case of barley^27^ and enhanced aluminum tolerance in maize^28^. Two genes (Ca_05989 in CDC Luna and Ca_14204 in CDC Corinne) that encode for NAC transcription factor showing higher CNVs were identified. Involvement of *CarNAC3* in drought stress response and its differential expression patterns during seed development and germination was reported in chickpea^29^,^30^. Nevertheless, NAC genes were found associated with pod shattering resistance in case of soybean^31^. CNV for two genes Ca_23345 and Ca_11443 that encode disease resistance were present only in CDC Luna variety, which is resistant to AB. Given the importance of early maturity, owing to large shift (~30% of total chickpea area) from cooler long season to short warmer season environments and increasing incidence of reproductive heat stress^20^, varieties like ICCV 92318, ICCV 92337, ICCV 95334 and ICCV 92311 were developed using ICCV 2 (carrying early flowering gene *efl*1 of known allelic relationship). Major flowering time QTLs were reported on the Ca4 using ICCV 2^32^, similarly Varshney and colleagues^33^ also reported on the Ca4 using ICC 4958. In the current study, we identified CNV in gene Ca_04471 that encodes for Leucine-rich repeat receptor-like kinase protein FLORAL ORGAN NUMBER1 (FON1), a putative ortholog of CLV-1 in Arabidopsis^34^. CLV-1 is one among the three genes in CLAVATA pathway. Kayes and Clark^35^ earlier reported that plants that lost any one of three genes from enlarged shoot apical meristems and floral meristems, result in increased number of flowers and floral organs. We identified high CNV for CapLEA-2 (Ca_13450) gene that encodes late embryogenesis protein-2, previously reported to be upregulated in drought stress tissues^36^. Interestingly, all varieties (RSG 888, RSG 44, Pusa 1105, IICV 95311, ICCC 37) containing CapLEA-2 were drought tolerant.

In terms of PAVs, we identified a total of 24,603 PAVs on the basis of genome coverage to the reference genome. Number of PAVs ranged from 2,736 to 13,821 in the 129 varieties. Further, we also observed variation in number of PAVs among varieties released in three different time periods, with large number of variations in RP3 followed by RP2 and RP1 (Table 1). Large number of PAVs were present on Ca4, both in the case of desi and kabuli as well as different time periods of release. Similarly, recent studies in other crops also indicated large variation in number and distribution of PAVs across the genome^37^,^38^,^39^. We considered a gene to be lost in a given variety if the coverage is <10% in the sample for that gene. Although, number of Indels were minimum in case of CDC Luna, the number of CNVs (1,508) and PAVs (3,148) were higher as compared to other varieties (Supplementary Table 7). Compared to the reference genome, a total of 458 genes (15.5% of all PAVs) were absent in one variety, while 191 genes (6.5%) were absent in two varieties, 134 (4.3%) in three cultivars, and 2,170 genes were missing in more than three varieties. GO enrichment analysis of PAV genes mainly grouped the variations to developmental, cellular and metabolic processes as well as response to stimulus (Supplementary Figure 6). Similarly, several PAV gene enrichment analysis earlier indicated the involvement of PAV genes in stress responses, especially the disease resistance^38^,^39^,^40^,^41^. Among 129 varieties, CSG 8962, a high yielding desi variety released for cultivation in NWPZ of India in 1997, had maximum number of PAVs (1415). *PHOTOPERIOD-INDEPENDENT EARLY FLOWERING1 (PIE1*) is an activator of the *FLOWERING LOCUS C (FLC*) gene, originally identified as a suppressor of FRIGIDA-dependent late flowering in Arabidopsis^42^. In the present study, we identified a PAV for Ca_14192 gene that has homology to *PIE1* and is absent in 92 chickpea varieties. Although we do not have phenotyping information on photoperiod sensitivity of these varieties, the chickpea genotypes originating from the tropics are largely short in growth duration and thus having a less degree of photoperiod sensitivity^43^. In addition, susceptibility of Blanco Lescho, a Spanish breeding line/cultivar, to AB can be attributed to presence of PAV in gene Ca_1095 that encodes for blight resistance protein (Supplementary Table 11).

### Temporal and genome diversity trends

Special attention has been focused in recent years to understand the temporal, spatial and genome diversity trends in crop plants. To understand the diversity at genome level we computed diversity statistics like nucleotide diversity (θ_π_) and Watterson’s estimator (θ_w_) among desi and kabuli varieties, in addition to three different release periods. The overall θ_π_ and θ_w_ in case of 129 varieties were 0.701 × 10^−3^ and 0.578 × 10^−3^ respectively (Supplementary Table 12), which is less than that of the cultivated soybean^37^. Nucleotide diversity in desi varieties (θ_π_ = 0.684 × 10^−3^) is higher compared to kabuli (θ_π_ = 0.650 × 10^−3^) varieties. This indicates higher recombination rate present in the desi group. The high diversity among varieties released in RP3 (θ_π_ = 0.684 × 10^−3^) can be attributed to involvement of multiple crosses while developing these varieties, which is evident from the pedigree information (Supplementary Table 1), compared to double or triple crosses in case of varieties of RP2 or predominantly direct selections from local collections or involving single or double crosses as in case of RP1. For instance, when we look at the pedigree, ICCV 95423 (developed from the cross (ICC 7676 × ICCC 32) × ((ICCC 49 × FLIP 82-1 C) × ICCV 3)), has ICC 7676, ICCC 32, ICCC 49, FLIP 82-1 C, ICCV 3 genotypes in the genetic background. Use of multiple genotypes or multi-parent advanced generation intercross (MAGIC) populations have been recently reported in several crops to enhance the diversity^44^. High level of diversity among varieties released in Canada, Australia can be attributed to the use of cross between desi × kabuli genotypes while developing the varieties. This study also re-emphasizes the use of multiple parental genotypes for harnessing the genetic diversity for crop improvement which has been adopted in several crop plants^44^.

**Figure 2:**
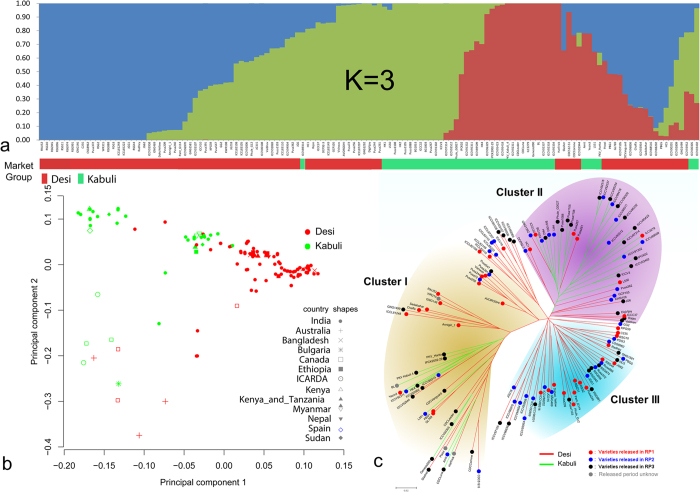
Genetic diversity and population structure in 129 chickpea varieties. (**a**) Population structure analysis using 1,378,790 SNPs with Frappe software^45^ clearly indicates three sub-populations, (**b**) Principal component analysis clearly demarcate the Canadian and Australian varieties, (**c**) Released varieties from three different periods (RP1 (before 1992), red circles; RP2 (between 1992 and 2002), blue circles and RP3 (after 2002), black circles) were grouped into three clusters (Cluster I, Cluster II and Cluster III). Clustering indicate the diversity among all three release periods and grouping of kabuli varieties with desi in Cluster I. Grouping of some desi with kabuli in Cluster II was due to inter crossing of desi and kabuli genotypes and vice versa for enhancing yield and disease resistance.

Using FRAPPE, which employs maximum likelihood method to determine population structure^45^, we identified three sub-populations (Fig. 2a). Admixture observed in individuals of sub-population explains involvement of different parental lines in developing these varieties. PCA analysis performed as per Patterson^46^, explains the extent of diversity among varieties released in different countries (Fig. 2b). In addition, genetic diversity analysis performed using neighbor joining (NJ) method further supports the results of structure as well as PCA analysis. NJ tree grouped 129 varieties into three major clusters (Fig. 2c) namely Cluster I, Cluster II, and Cluster III. Cluster I and Cluster II predominantly consist of varieties released in RP3, while Cluster III is dominated by varieties released in RP1 (Fig. 2c). Cluster III exclusively comprised of desi varieties, while Cluster II predominantly contained kabuli varieties and Cluster I predominantly contained desi varieties. Among four sub-clusters, Cluster I, Cluster Ib, Cluster Ic and Cluster Id comprised of desi varieties, while Cluster Ia comprised of both desi and kabuli varieties. The varieties grouped in Cluster Ia were found more diverse, owing to use of different parental lines while developing or majority of them are single plant selections from a cultivar or breeding lines. Similarly, majority of varieties (50%) grouped in Cluster Ib were either breeding lines or selections from local landraces or germplasm collections. We observed seven high yielding varieties (four early, one medium and two late) developed at Indian Agricultural Research Institute (IARI), India released for cultivation in NWPZ, NEPZ and Central Zone grouped together in Cluster Ic had at least one parent common in their genetic backgrounds. For instance, 850-3/27 is one of the common parents for Pusa 244 and Pusa 256 released in 1985, while Pusa 256 is in the genetic background of two varieties Pusa 391 and Pusa 72, nevertheless, Pusa 547 is a mutant line derived from Pusa 256 (BG 256). Interestingly, varieties released in Bangladesh (3), Ethiopia (3), Kenya and/or Tanzania (2), Nepal (1) were grouped in the Cluster Id and all these varieties had K-850 or GW-5/7 as one of the parental lines common in their pedigree. Majority of varieties grouped in Cluster III (90%) were released in India, except two varieties (ICCV 88202/Sona, Genesis 836) released in Australia, one in USA (Myles), one in both Kenya and Tanzania (ICCV 97105). Grouping of kabuli varieties like PKV Harita, Yezin 3 in the Cluster I along with desi varieties and similarly grouping desi varieties like HC-1, GNG 1292, DCP 92-3 in the Cluster II along with kabuli varieties is due to use of cross between desi × kabuli or kabuli × desi genotypes that were extensively made to exploit the useful genes from one group to the other, which is evident from the pedigree information (Supplementary Table 1).

### LD decay and haplotype patterns in disease resistance genes

We computed the decay of LD along the physical distances among varieties released in RP1, RP2 and RP3 as well as on all 129 varieties. Interestingly, the overall LD in case of 129 varieties reached its maximum at 290 kb and varied among different chickpea pseudomolecules (Supplementary Figure 7). However, LD decay was rapid in the case of RP1 (230 kb) followed by RP3 (250 kb) and RP2 (260 kb) (Supplementary Figure 8). We also looked for differences in the pattern of haplotype blocks between the desi and kabuli varieties, estimated the haplotype blocks using PLINK software^47^, which adopted the same methodology as in Haploview^48^. We identified ~36,000 haplotype blocks in desi varieties and ~38,000 haplotype blocks in kabuli varieties. The longest haplotype blocks observed were 174 kb and 232 kb in length in the case of desi and kabuli varieties, respectively. Interestingly, a large number of smaller haplotype blocks (size < 1 kb) were observed in kabuli varieties compared to desi varieties (30,339 haplotype blocks in kabuli varieties; 26,926 haplotype blocks in desi varieties) (Supplementary Figure 9). The density of the SNPs may have an inverse effect on the haplotype block length. This analysis will be useful to identify loci associated with economically important traits as reported earlier in case of soybean^49^. Further, the elucidation of haplotype block structure can bring important considerations for genome-wide association studies (GWAS) and genomic selection studies^50^. For instance, a set of most informative SNPs can be selected from several SNPs in a haplotype block, thus optimizing the design and analysis of GWAS. Nevertheless, haplotype blocks can also be used for detection of genomic regions under selection during evolution, and identification of signatures of recent positive selection during crop breeding.

### Signatures of positive selection for yield

**Figure 3:**
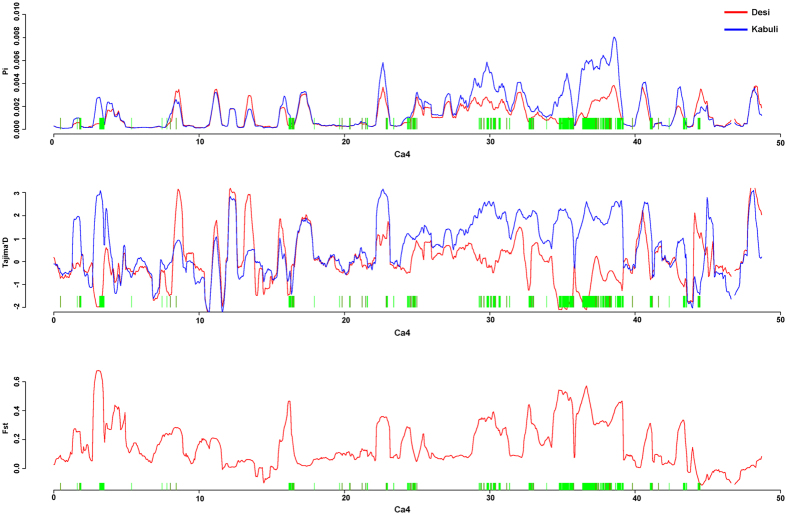
Signatures of positive selection on Ca4 pseudomolecule that carries haplotypes for drought tolerance. We identified five genomic regions >150 kb on Ca4 with significant negative Tajima’s *D* in desi varieties and two regions with significant negative Fu and Li’s D*. While in case of kabuli varieties, we identified one genomic region each on Ca4 and Ca3 with significant negative Tajima’s *D (*Supplementary Table 14).

The substitution ratio between non-synonymous and synonymous substitutions per site is often used to identify evidence for adaptive evolution^51^. In the present study, all varieties developed possessed dS/dN ratio >1 indicating positive selection for different traits across all three release periods (Supplementary Table 4). Further, we chose the highest *F*_*ST*_ region and highest differences of π region (overlap) as the differentiation between desi and kabuli and identified 310 regions which is equaling to ~2% (14.12 Mb) of chickpea genome and maximum number of regions were identified on Ca4 (Fig. 3). We call these regions as signatures of selection during improvement. We identified five genomic regions >150 kb on Ca4 and one region on Ca3 with significant negative Tajima’s *D* found in desi varieties and two regions with significant negative Fu and Li’s D*. While in the case of kabuli, we identified one genomic region each on Ca4 and Ca3 with significant negative Tajima’s *D (*Supplementary Table 13). Three SNPs Ca4_29886089 (between 29.77–29.99 Mb), Ca4_34891815 (between 34.76–34.94 Mb) and Ca4_35659037 (between 35.51–35.78 Mb) present in SPS regions on Ca4 were significantly associated with 100 seed weight under both rainfed and irrigated conditions and in more three seasons indicating these three regions on Ca4 are signatures of positive selection for enhancing chickpea yields. In addition, we also identified seven regions on Ca4 with positive Tajima’s D value which indicate these regions might have undergone balancing selection during breeding (Supplementary Table 14). Ca_14192 gene that encodes for early flowering i.e., *efl1* gene^52^ is present in one of the seven regions of balancing selection.

## Discussion

Given the importance of development of climate smart crops, understanding the temporal and genome diversity trends among varieties of chickpea will enable designing future breeding strategies. In this direction, after sequencing 100 varieties in this study, and together with re-sequencing data for 29 varieties available from the earlier study, we analyzed genomes of 129 varieties released between 1948 and 2012. These analyses have provided opportunities to inspect the genetic and genomic changes reflecting the history of breeding, which we call as breeding signatures (SNPs, indels, CNVs and PAVs). We detected 310 regions spanning 2% of the chickpea genome that had been differentially selected between varieties released in RP1, RP2 and RP3. These regions include large number of known functional genes and loci associated with important agronomic traits like early flowering, disease resistance previously reported using both linkage and LD based trait mapping approaches^4^,^6^,^52^. Identified CNVs and PAVs will become increasingly relevant for the chickpea breeding community as crop production expands to short hotter regions with poor soil moisture and accumulation of toxic metals and chemicals over years of intensive agriculture. The mounting evidence that CNV gains in specific genes can have tremendous effects on abiotic stress tolerance^52^. Higher nucleotide diversity observed among varieties in RP3 indicated an increase in diversity in the primary gene pool as result of recent chickpea breeding programs. Although, recent studies reported modern breeding processes had no significant change in diversity in several crop species^53^,^54^,^55^. The number of selected haplotypes may serve as an indicator for evaluating the breeding potential of varieties to guide more efficient selection. A variety accumulating a whole complement of selected haplotypes might be an “ideotype” at the genomic level, which may be of both high-yielding and good adaptation to broad environmental conditions. In summary, we unraveled the genome architecture of 129 varieties representing all five global chickpea research domains. Our study paves the way to harness the existing diversity and enables prioritizing low diversity regions that require introgression of foreign alleles for further improvement.

## Methods

A total of 100 varieties released in 14 chickpea growing countries were re-sequenced on Illumina HiSeq^TM^ 2000 sequencing machine using WGRS approach (Supplementary Table 1).

### Variant detection

To ensure quality, adaptor sequences were trimmed from raw data of 129 varieties (100 from this study and 29 varieties sequenced earlier) and reads containing > 20% low quality bases (quality value ≤ 7) and reads with > 5% “N” nucleotides were removed^56^. Clean reads were mapped on to the reference genome of chickpea using SOAP2^57^ (parameters were -m 100 -x 800 -s 35 -l 32 -v 3 -p 4). We then used SOAPsnp3 to calculate the likelihood of all possible genotypes for each sample (default parameters were used). To identify the potential variants, aligned data from all the genotypes were combined and targeted the regions where the overall depth ranges from 78 to 2500. All individual likelihood files were then integrated to produce a pseudo-genome for each site by maximum likelihood estimation followed by filtering using criteria that included copy number (≤1.5), sequencing depth (according to average depth of each accession) and quality. SNPs that passed above criteria were included in the final SNP set. Assignment of genotypes to individual was according to the final SNP set, which base types were allocated back to each individual depending on genotypes of the final SNPs and each individual likelihood file. Filtered the SNPs with individual missing ratio reach up to 50%. The loci with estimated allele frequency not equal to 0 or 1 were determined as SNPs.

For insertion and deletion identification, reads were mapped to the reference genome^14^ using SOAPaligner with a parameter “–g 10”, which allows maximum of 10 bp gap within the hit of one single read. Small insertions and deletions (1–10 bp), referred as Indels, were identified using SOAPindel^24^. For identification of SVs, we used the pair-end mapping information for identifying reads with abnormal insert sizes, or only one end mapped to the genome and determined a SV when more than 3 paired reads abnormally mapped. This method was implemented in the SOAPsv^58^.

We identified CNVs according to the mapping depth described previously^23^. Assuming a Poisson’s distribution of sequencing depth, the genome regions were divided into initial windows, where the sequencing depth didn’t differ significantly. The mean depth of each window was then calculated and compared to nearby windows. Initial windows were thus further merged if there were no significant depth differences in the nearby initial windows. Merging of the window process was repeated once more, thus the edges and the copy number of each window was decided. As we detected lost genes later, we only retained CNVs that had more copies than the reference genome (copy number >1).

We filtered the identified Indels to obtain the PAVs. For a deletion region, if the average sequencing depth was less than 10% of the genome-wide average sequencing depth, we determined this sample to have absence variation in this region. Similarly for an insertion region, if the average sequencing depth was more than 50% of the genome-wide average sequencing depth, we determined this sample to have presence variation in this region. For the sub-populations or groups, if more than three samples consistently had PAV at one region, this sub-population or group was determined to have this PAV.

### Population structure and genetic relationships

We conducted population structure analysis using the Frappe software^45^. We ran 10,000 iterations, and the number of clusters (K) was set to 2 to 7. We used the final SNP dataset with 1,378,790 SNPs for conducting PCA as described earlier^46^. Majorly, the eigenvector decomposition of the transformed genotype data was performed using the R function eigen, and the significance of the eigenvectors was determined with a Tracey-Wisdom test, implemented in the program twstats, provided by the EIGENSOFT software^59^. We used the final SNP dataset to construct the phylogenetic tree. All SNPs were used to calculate the genetic distances between different accessions following the procedure previously described^22^. Then, the neighbor-joining method in the software PHYLIP^60^ was used to construct the phylogenetic tree according to the distance matrix. Finally, MEGA4^61^ was used to display the phylogenetic tree.

### LD decay and gene loss analysis

We determined LD decay at whole genome level as well as on each pseudomolecule in case of varieties released in three different release periods (RP1, RP2 and RP3) and also among desi and kabuli chickpea varieties employing Haploview^48^. We calculated diversity parameters like θπ, θω, Tajima’s D as described earlier^14^.

## Additional Information

**Accession codes:** The raw sequence reads are submitted to NCBI SRA. Bioproject ID: PRJNA328187.

**How to cite this article**: Thudi, M. *et al*. Recent breeding programs enhanced genetic diversity in both desi and kabuli varieties of chickpea (*Cicer arietinum* L.). *Sci. Rep.* **6**, 38636; doi: 10.1038/srep38636 (2016).

**Publisher’s note:** Springer Nature remains neutral with regard to jurisdictional claims in published maps and institutional affiliations.

## Supplementary Material

Supplementary Figures

Supplementary Tables

## Figures and Tables

**Table 1:** Genome-wide variations among 129 chickpea genotypes.

	SNPs				Indels				CNVs	PAVs
	Intra-genic		Intergenic	Total	Intra-genic		Intergenic	Total		
	Exon	Intron			Exon	Intron				
All 129 genotypes	46,387	115,360	1,217,043	1,378,790	1,229	17,222	132,989	151,440	3,822	24,603
**Market type** (129 genotypes)	**46,387**	**115,360**	**1,217,043**	**1,378,790**	**1,229**	**17,222**	**132,989**	**151,440**	**3,822**	**24,603**
Desi (88 genotypes)	44,859	110,908	1,167,349	1,323,116	1,154	16,399	126,493	144,046	2,954	23,160
Kabuli (41 genotypes)	38,964	94,971	1,016,430	1,150,365	808	11,482	90,746	103,036	3,273	21,706
**Year wise** (124 genotypes)	**46,309**	**115,007**	**1,212,774**	**1,374,090**	**1,211**	**17,107**	**131,928**	**150,246**	**3,811**	**24,249**
Before 1993 (38 genotypes)	39,789	97,459	1,035,388	1,172,636	984	14,476	108,089	123,549	2,315	20,734
1993-2002 (40 genotypes)	40,380	99,195	1,055,415	1,194,990	810	11,412	91,292	103,514	2,318	21,150
2003-2012 (46 genotypes)	42,210	104,952	1,095,817	1,242,979	861	12,386	99,123	112,370	3,511	22,045

CNVs = Copy number variations; PAVs = Presence absence variations.
