# Supplementary material for: Recent breeding programs enhanced genetic diversity in both desi and kabuli varieties of chickpea (Cicer arietinum L.)
Source: Sci Rep. 2016 Dec 16;6:38636. doi: 10.1038/srep38636 (PMC5159902; doi:10.1038/srep38636)
Supplement: Supplementary Figures [file srep38636-s1.doc]

**Recent breeding programs enhanced genetic diversity in both desi and kabuli varieties of chickpea (*Cicer arietinum* L.)**

Mahendar Thudi^1,&^, Annapurna Chitikineni^1,&^,   Xin Liu^1,&^, Weiming He^2^, Manish Roorkiwal^1^, Wei Yang^2^, Jianbo Jian^2^, Dadakhalandar Doddamani^1^, Pooran M. Gaur^1^, Abhishek Rathore^1^, Srinivasan Samineni^1^, Rachit K. Saxena^1^, Dawen Xu^2^, Narendra P. Singh^3,4^, Sushil K. Chaturvedi^4^, Gengyun Zhang^2^, Jun Wang^2^ , Swapan K. Datta^5^, Xun Xu^2,*^, Rajeev K. Varshney^1,*^

^1^International Crops Research Institute for the Semi-Arid Tropics (ICRISAT), Hyderabad, India

^2^Beijing Genomics Institute (BGI) - Shenzhen, China

^3^All India Coordinated Research Project on Chickpea (AICRP), Indian Council of Agricultural Research (ICAR), New Delhi, India

^4^Indian Institute of Pulses Research (IIPR), Indian Council of Agricultural Research (ICAR), Kanpur, India

^5^Visva-Bharati, Santiniketan, India

**^&^Contributed equally- joint first authors**

*Corresponding authors: [r.k.varshney@cgiar.org](mailto:r.k.varshney@cigar.org); [xuxun@genomics.org.cn](mailto:xuxun@genomics.org.cn)


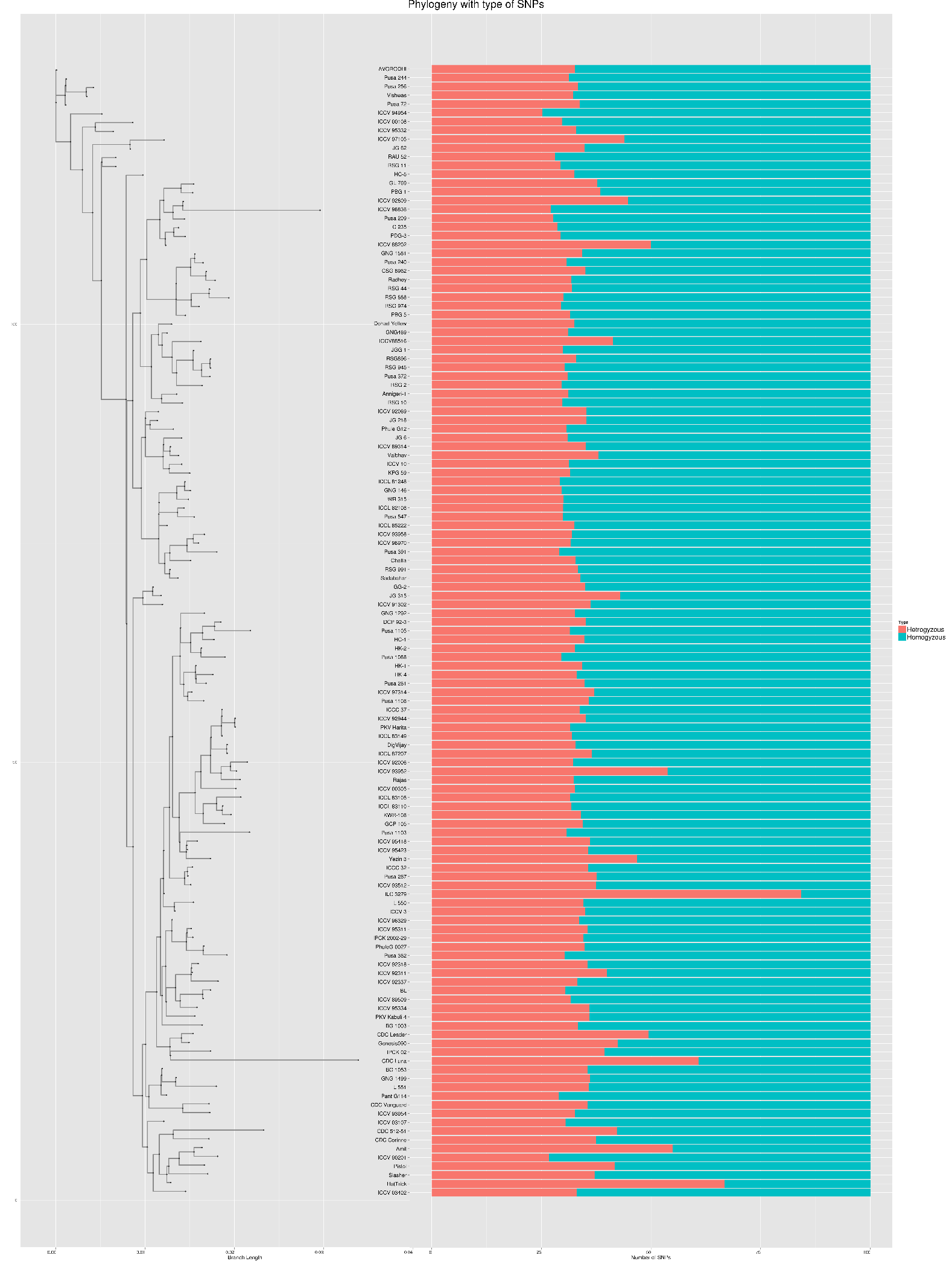


**Supplementary Figure 1: Number of homozygous and heterozygous SNPs in each chickpea genotype**


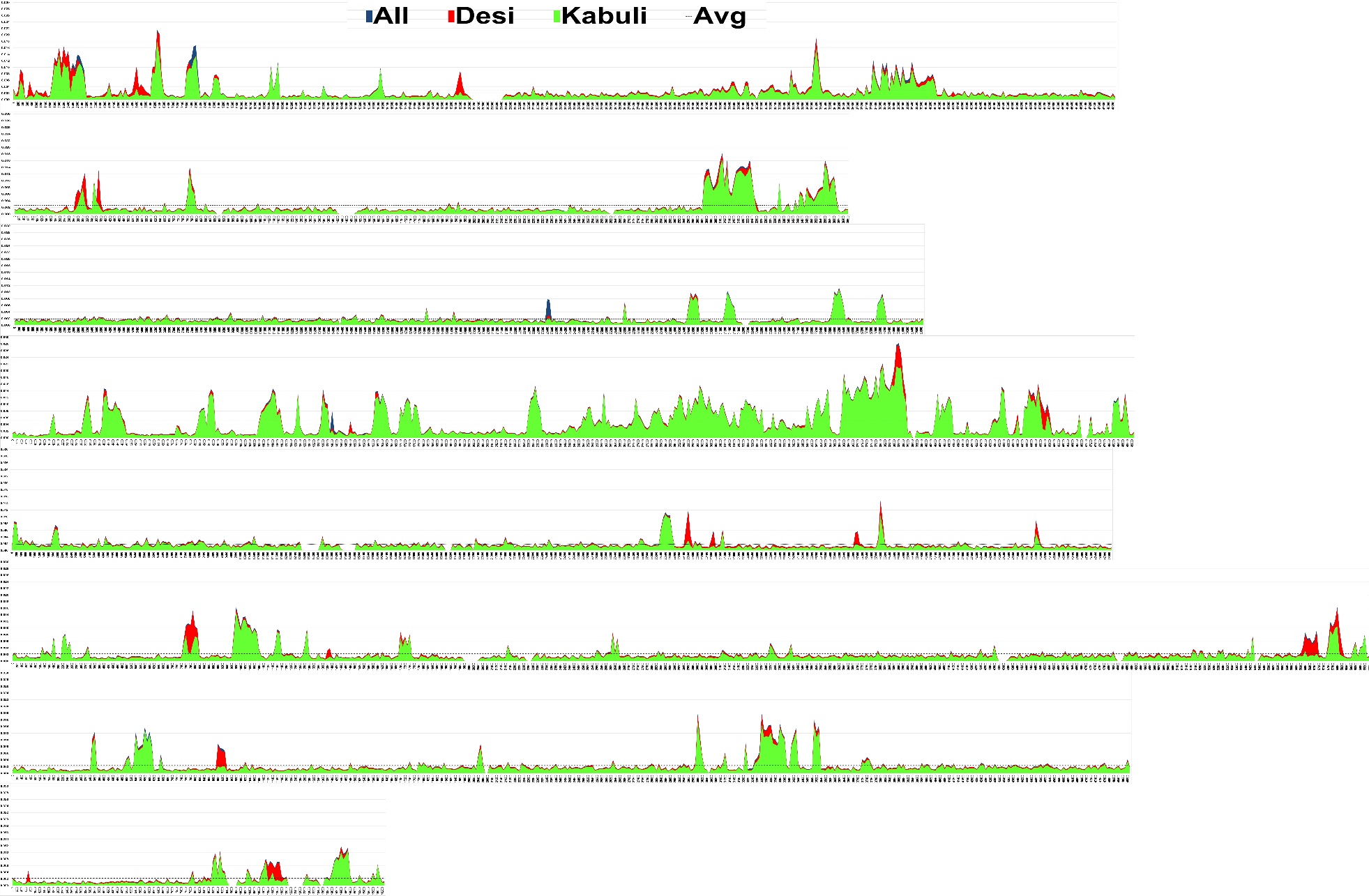


**Supplementary Figure 2: SNP density across on all eight pseudomolecules of chickpea**


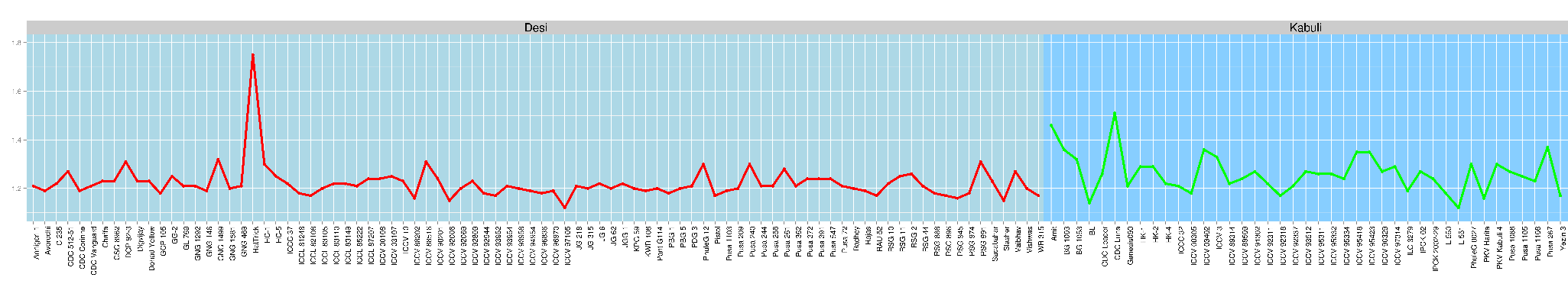


**Supplementary Figure 3: ds/dN ratio in all 129 chickpea genotypes**


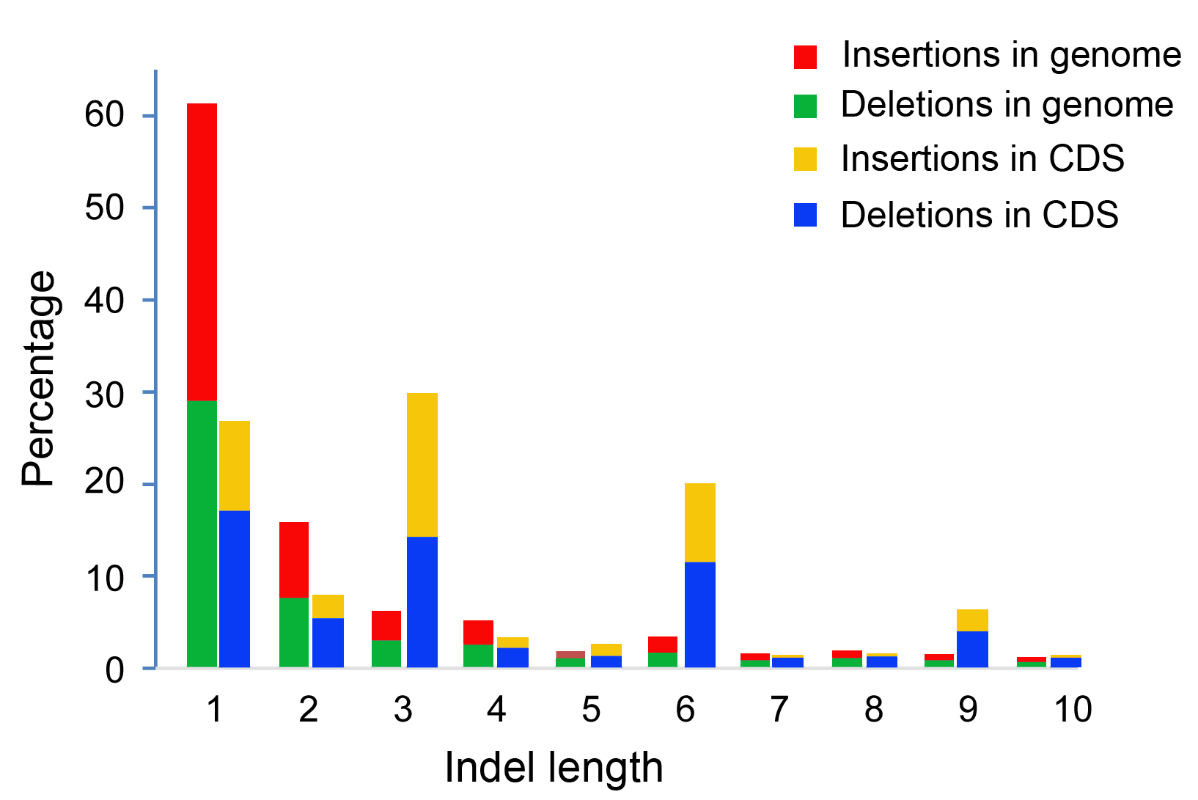


**Supplementary Figure 4: Proportion of Indels in genomic and CDS regions**


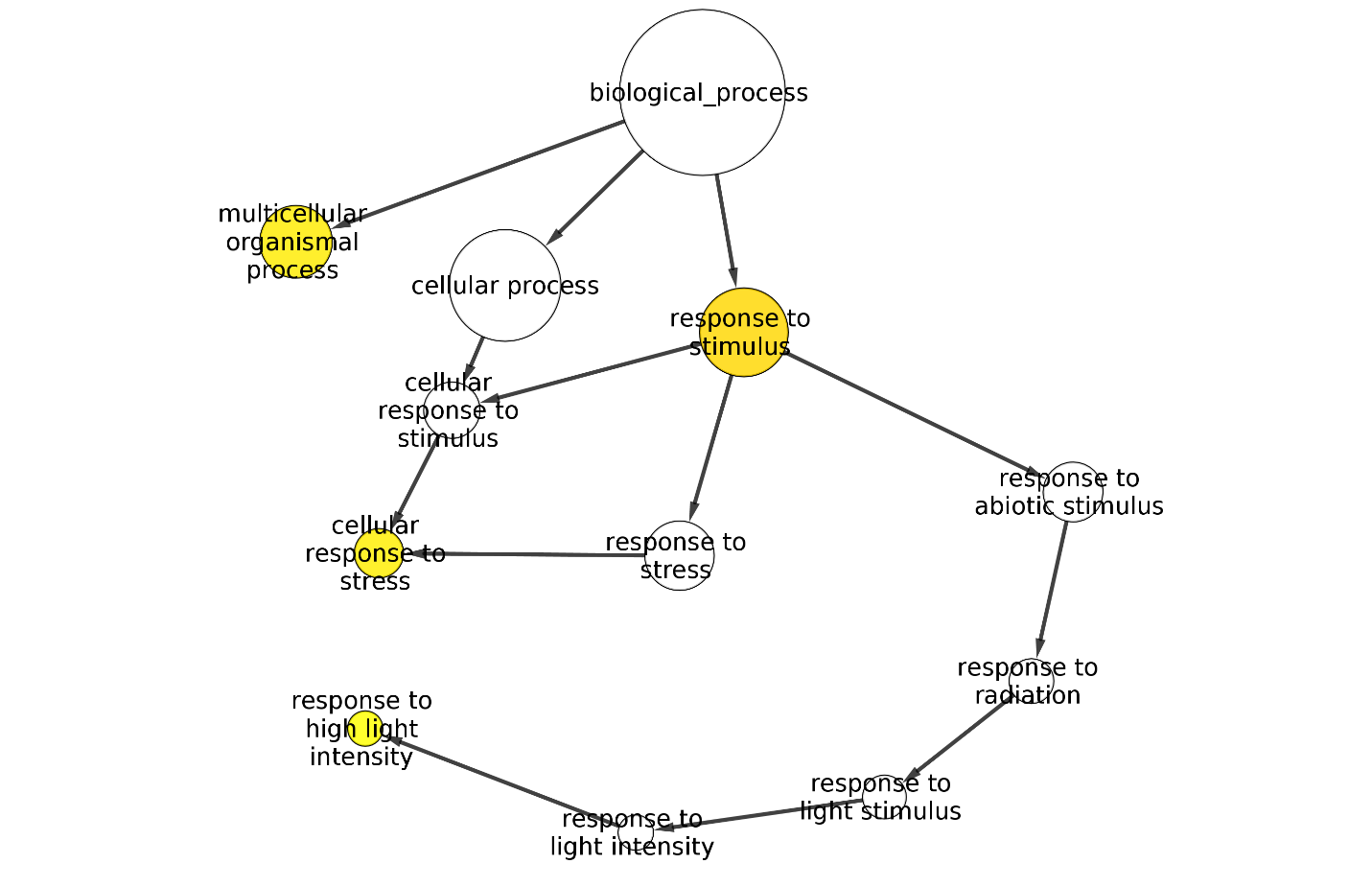


**Supplementary Figure 5: Gene enrichment analysis of CNVs indicate, majority of the genes are related to response to stimulus.**


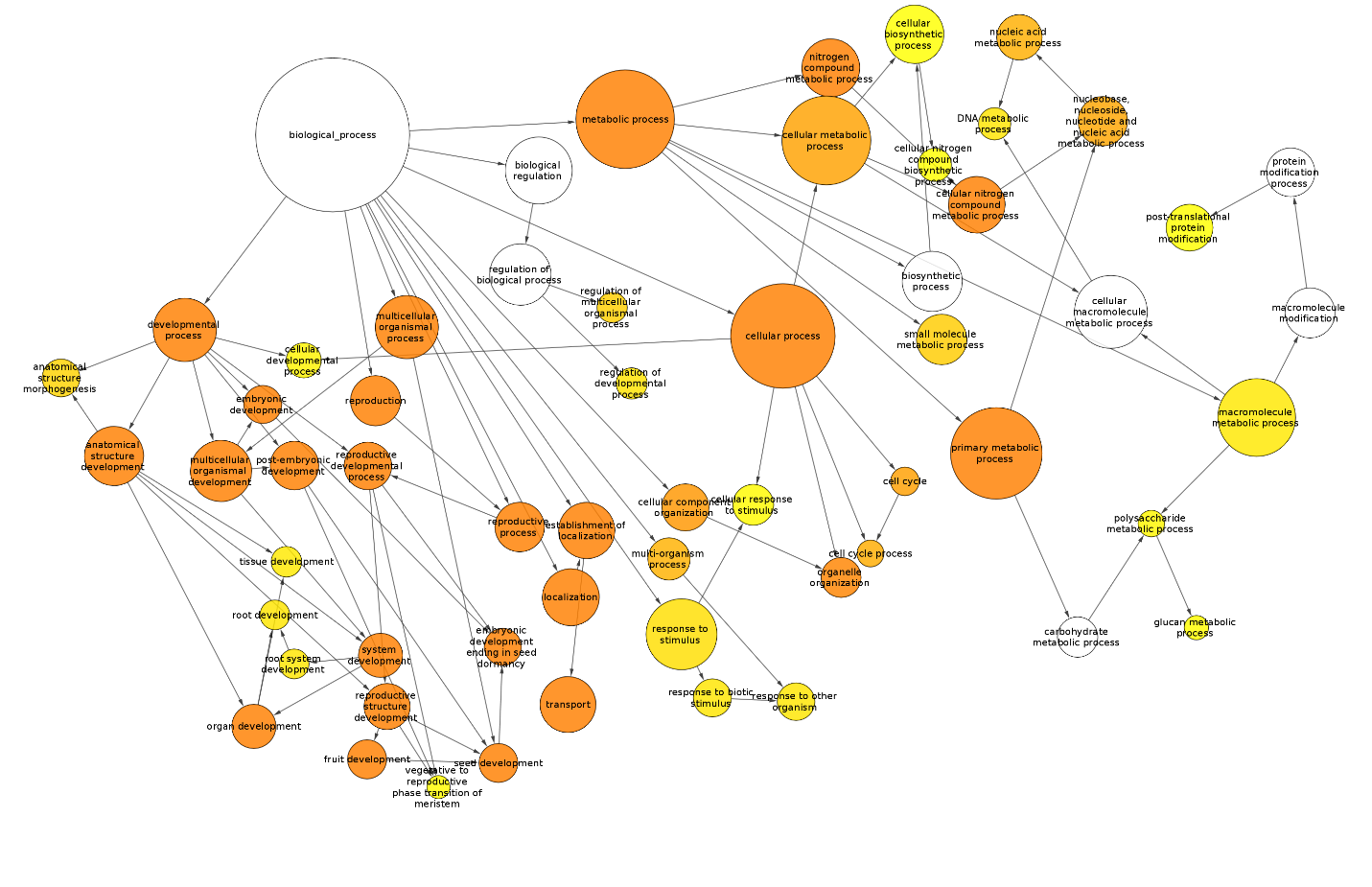


**Supplementary Figure 6: Gene enrichment analysis of PAVs indicate, majority of the genes are related to metabolic processes.**


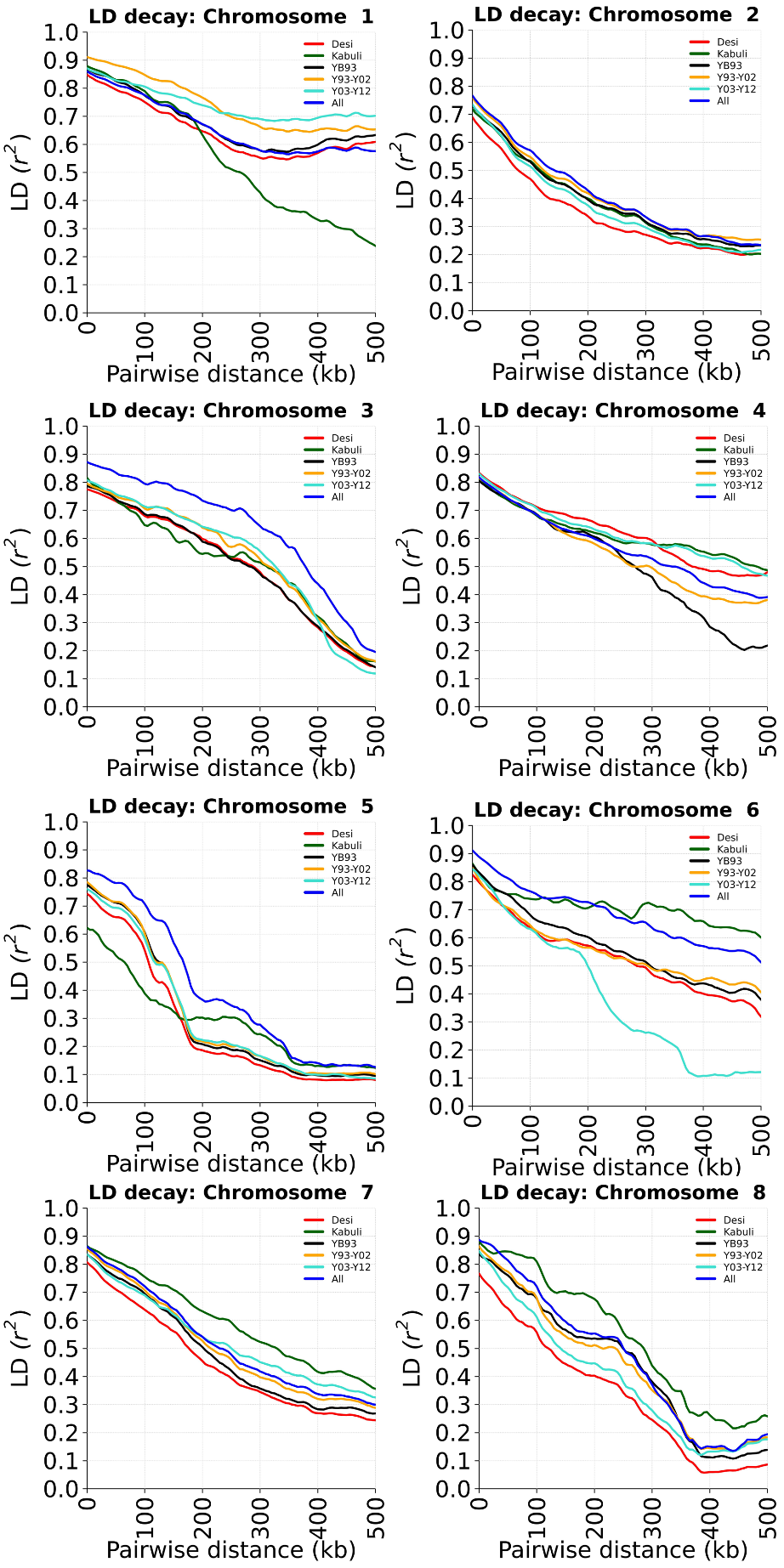


**Supplementary Figure 7: Chromosome wise LD decay in varieties released in RP1, RP2 and RP3 as well as across desi and kabuli genotypes.**


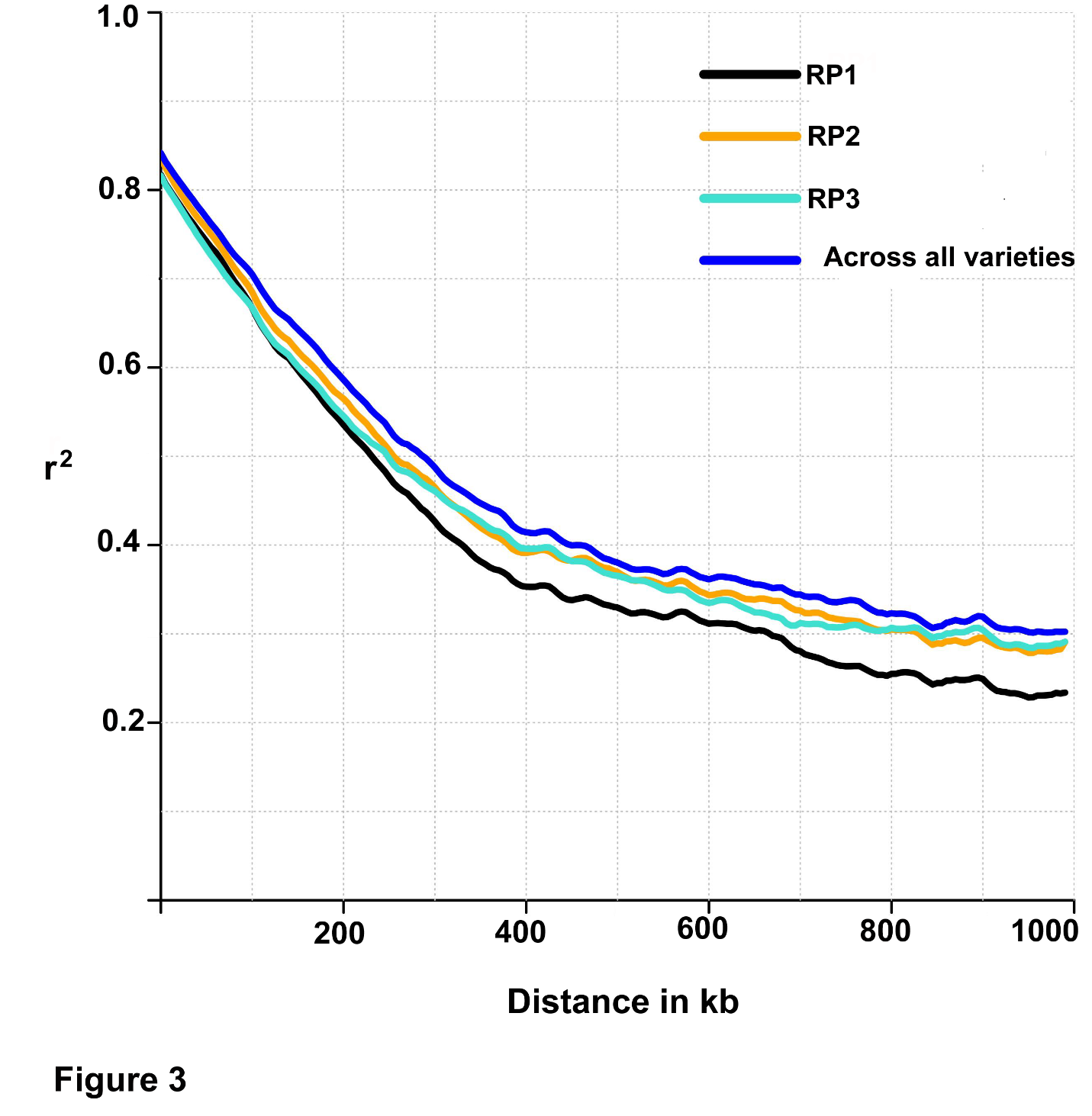


**Supplementary Figure 8: Linkage disequilibrium decay in released 129 chickpea varieties.**

LD decay was estimated in four groups namely varieties released before 1993 (RP1), varieties released between 1993-2002 (RP2), varieties released after 2002 (RP3) and across all release varieties. LD decay is rapid in the case of RP1 (230 kb) followed by RP3 (250 kb) and RP2 (260 kb).


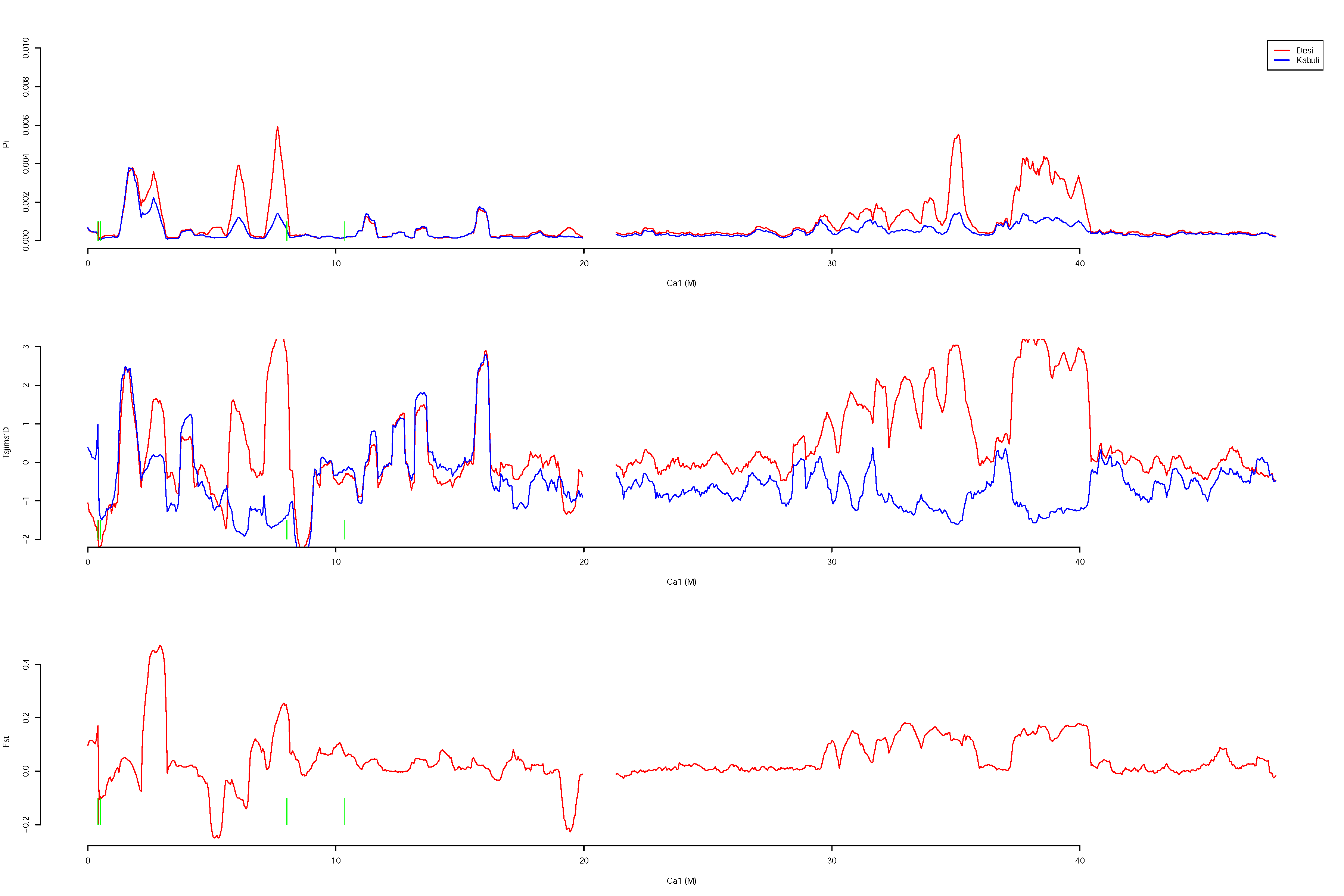


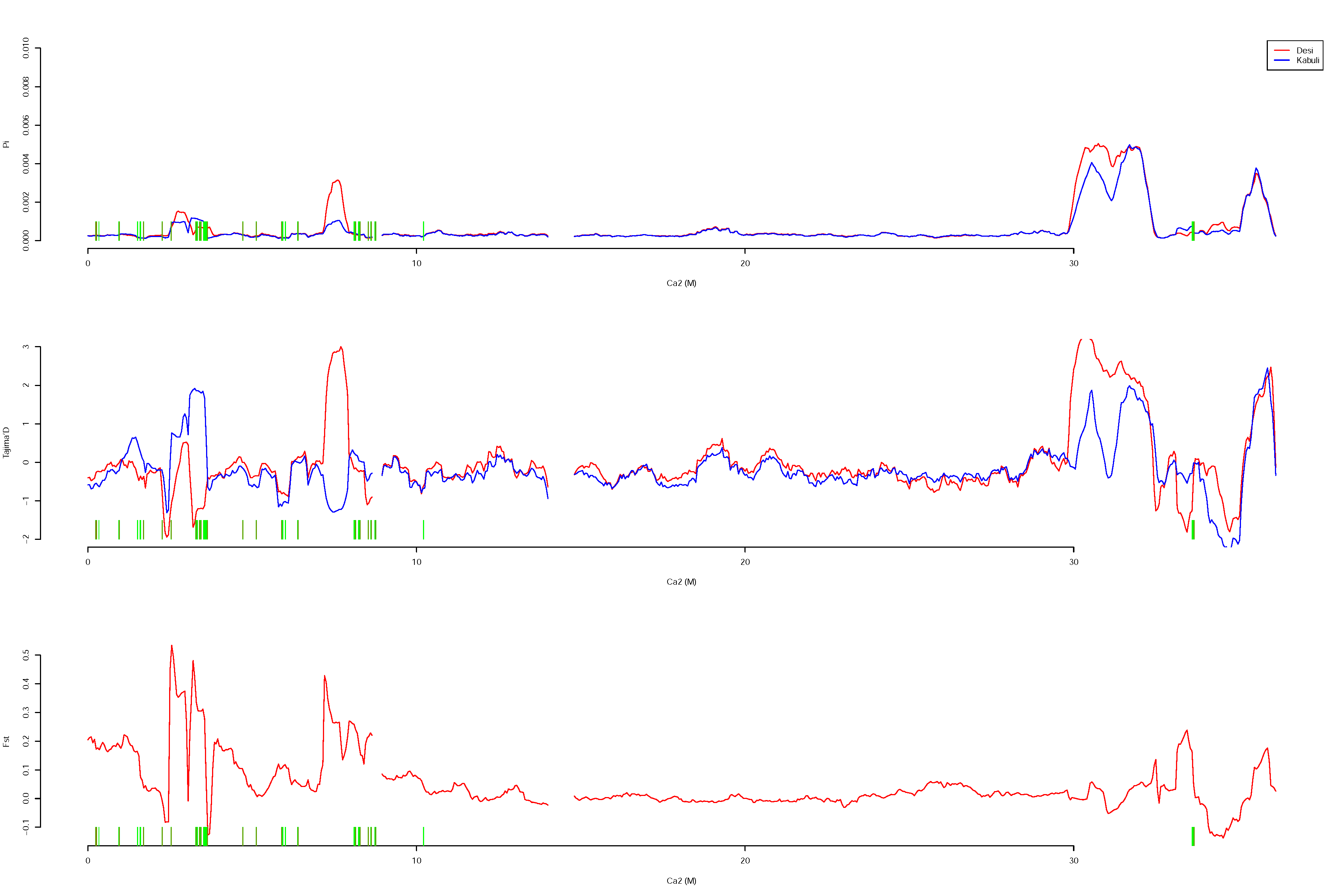


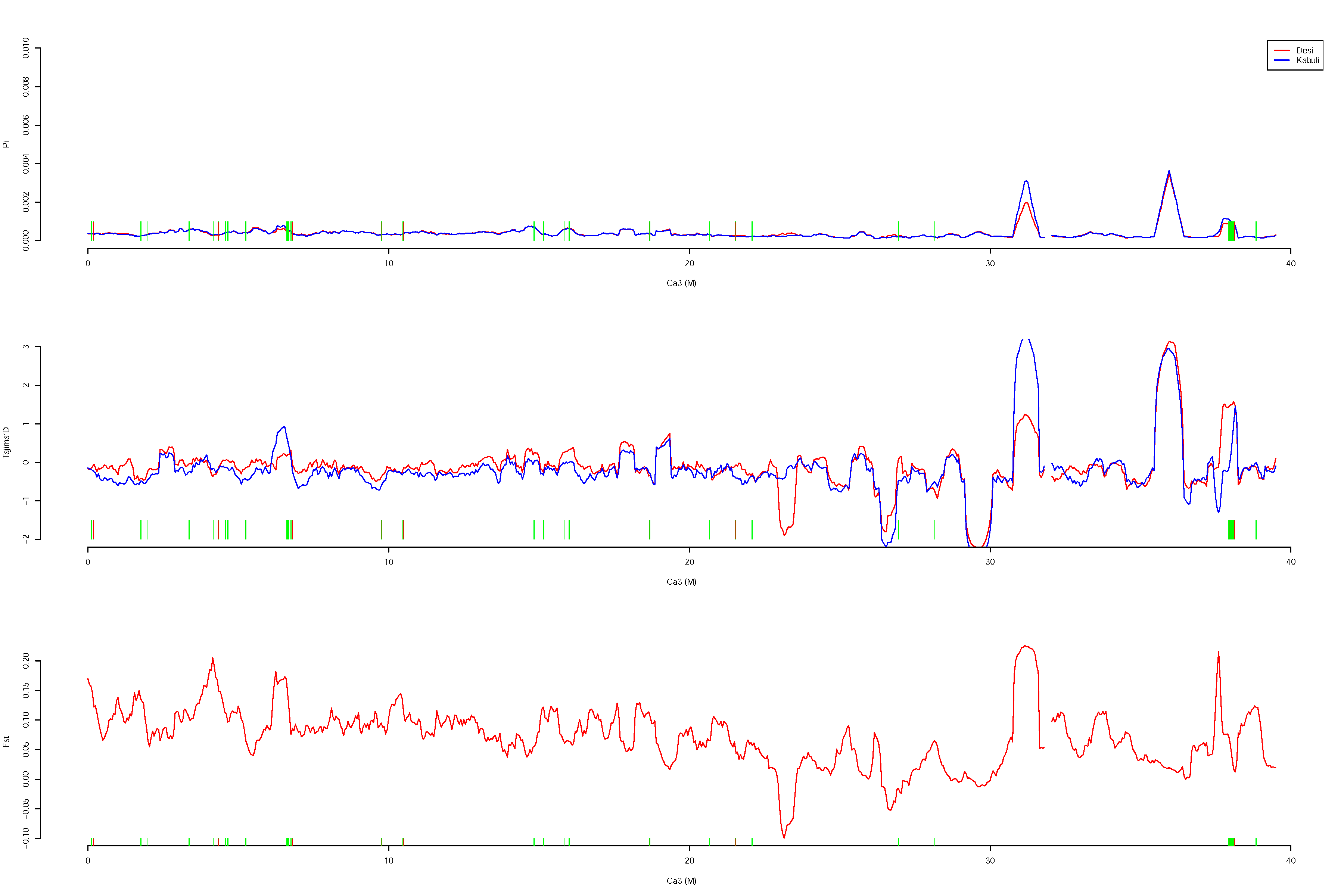


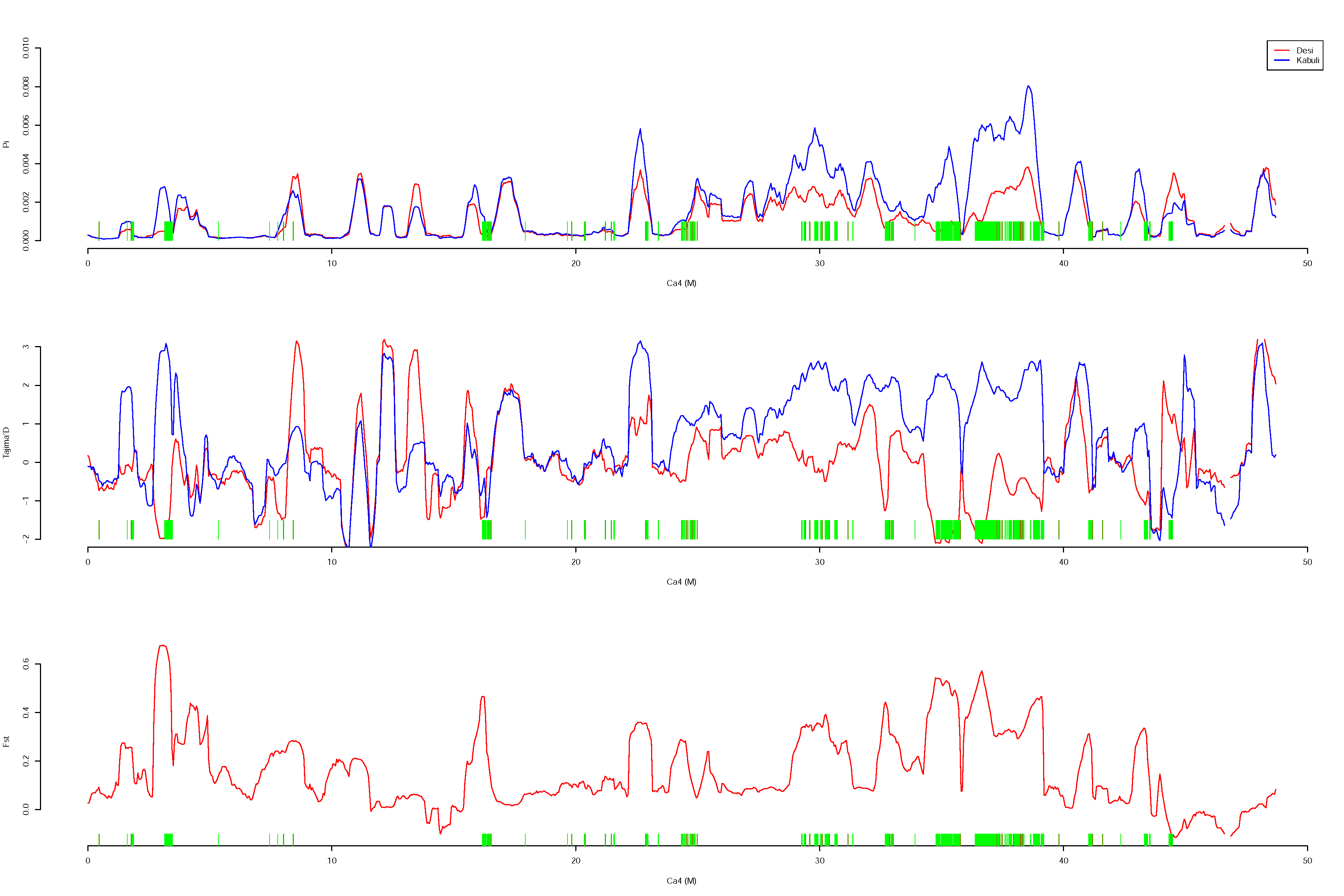


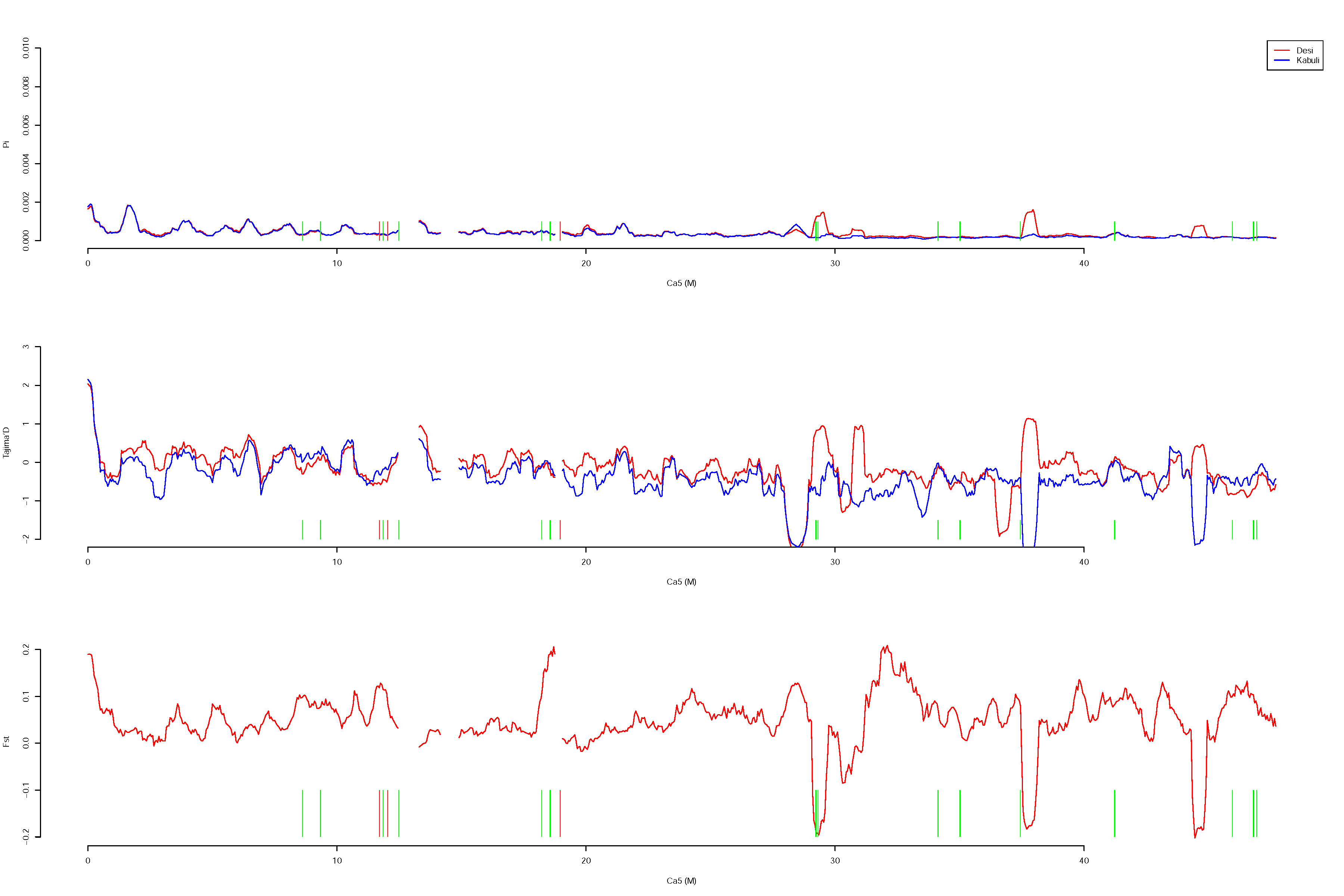


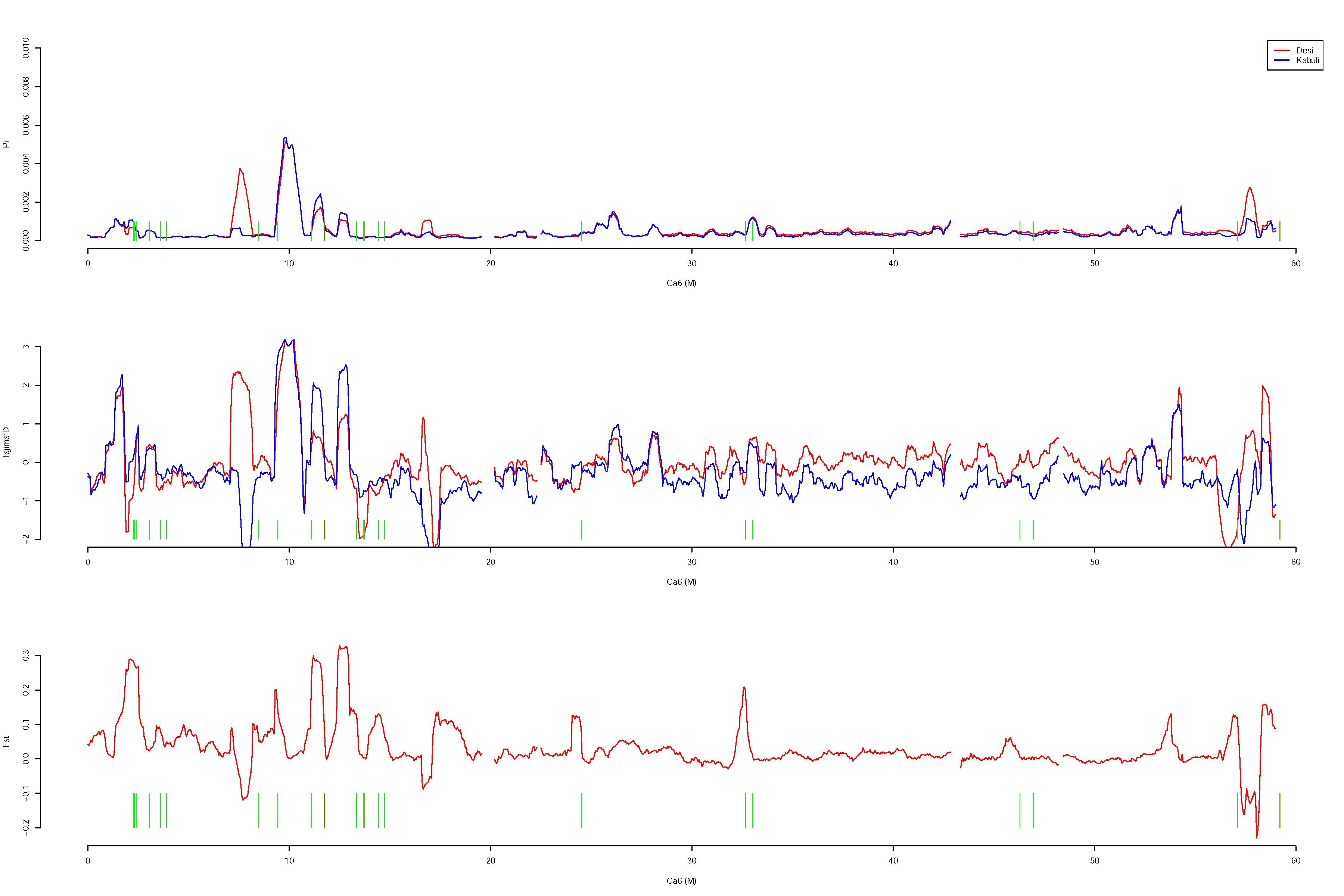


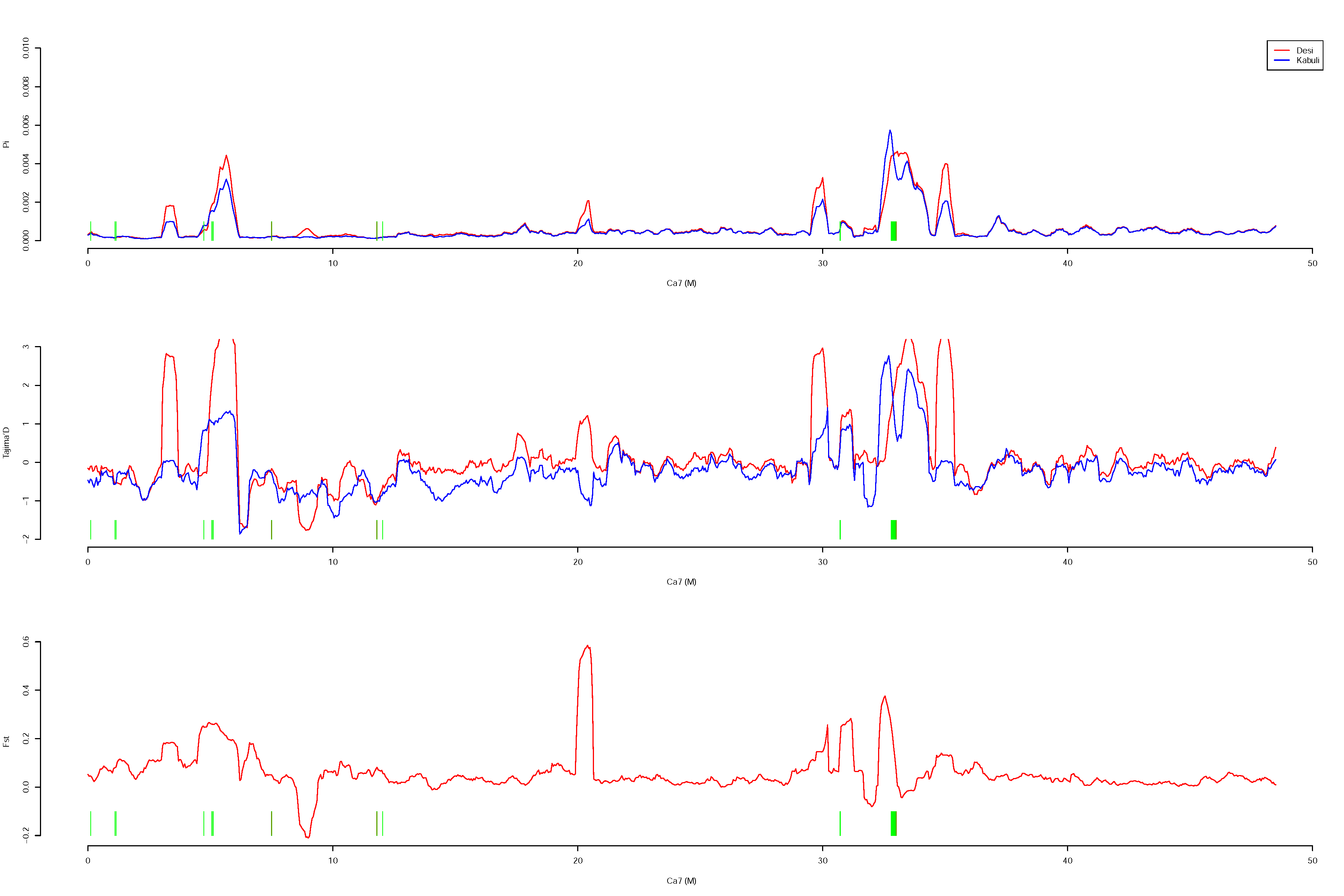


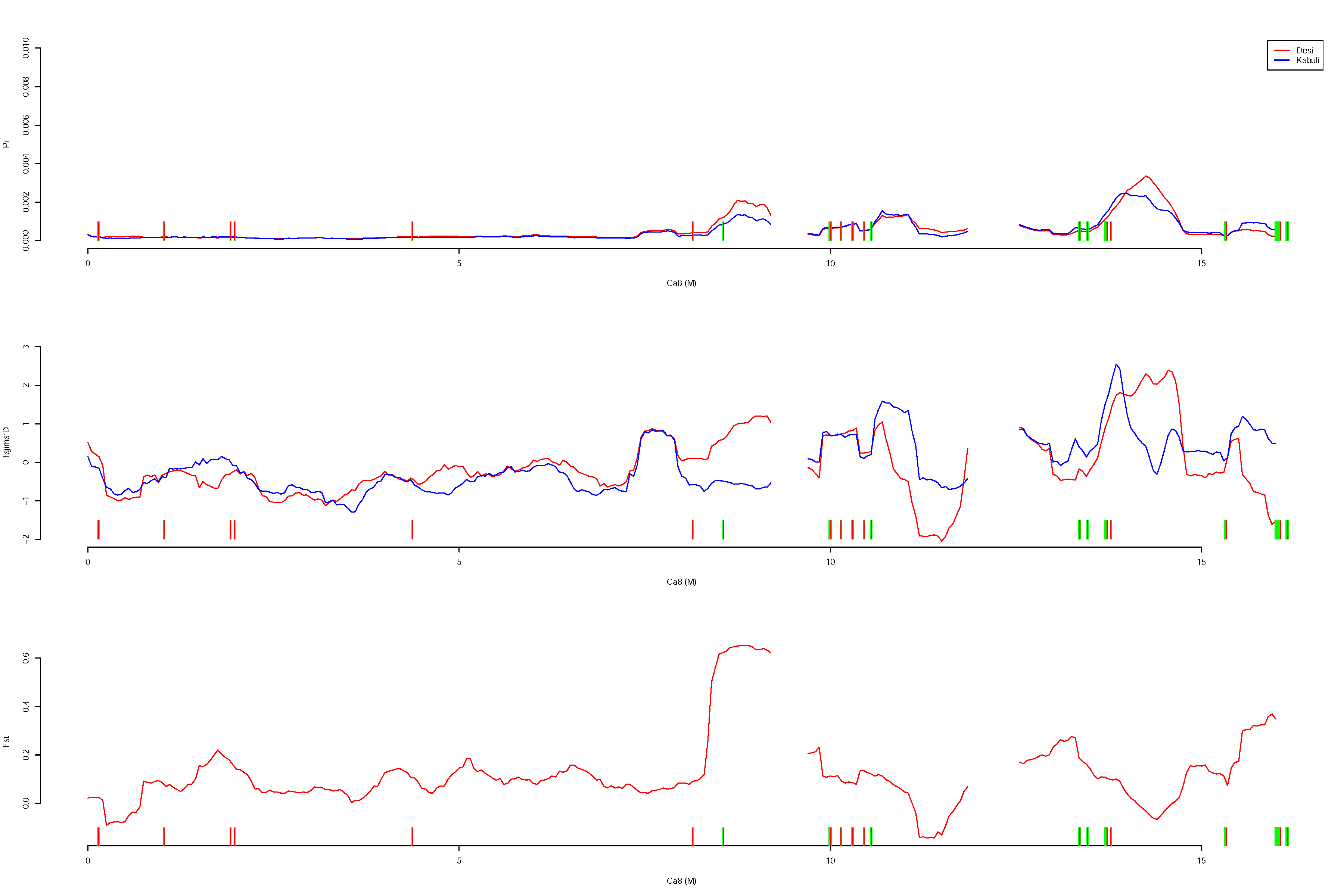


**Supplementary Figure S9: Haplotype blocks identified on all eight pseudomolecules**
